# From Farmworkers to Urban Residents: Mapping Multi-Class Pesticide Exposure Gradients in Morocco via Urinary Biomonitoring

**DOI:** 10.3390/jox15040120

**Published:** 2025-07-23

**Authors:** Zineb Ben Khadda, Andrei-Flavius Radu, Souleiman El Balkhi, Fagroud Mustapha, Yahya El Karmoudi, Gabriela Bungau, Pierre Marquet, Tarik Sqalli Houssaini, Sanae Achour

**Affiliations:** 1 Laboratory of Epidemiology and Research in Health Sciences, Faculty of Medicine and Pharmacy, Sidi Mohammed Ben Abdellah University, Fez 30070, Morocco; benkhadda.zaynab@gmail.com (Z.B.K.); tarik.sqalli@usmba.ac.ma (T.S.H.); 2 Doctoral School of Biomedical Sciences, University of Oradea, 410087 Oradea, Romania; 3 Department of Psycho-Neurosciences and Recovery, Faculty of Medicine and Pharmacy, University of Oradea, 410073 Oradea, Romania; 4 Department of Pharmacology, Toxicology and Pharmacovigilance, CHU Limoges, 87000 Limoges, France; souleiman.elbalkhi@chu-limoges.fr (S.E.B.); pierre.marquet@unilim.fr (P.M.); 5 P&T, UMR1248, Inserm, University Limoges, 87032 Limoges, France; 6 Pharmacology-Toxicology and Pharmacovigilance Department, Centre de Biologie Et de Recherche en Santé (CBRS), 2, Av. Martin Luther King, 87042 Limoges, France; 7 Department of Agronomy, National School of Agriculture, Meknes 50001, Morocco; mfagroud@gmail.com; 8 Laboratory of Ecology, Systematics, and Conservation of Biodiversity, LESCB URL-CNRST N° 18, FS, Abdelmalek Essaadi University, Tetouan 93002, Morocco; yahyaelkarmoudi@gmail.com; 9 Department of Nephrology, University of Hospital Hassan II, Fez 30050, Morocco; 10 Laboratory of Pharmacology and Toxicology, University Hospital Hassan II, Fez 30050, Morocco; sanae.achour@usmba.ac.ma; 11 Biomedical and Translational Research Laboratory, Faculty of Medicine and Pharmacy of the Fez, University of Sidi Mohamed Ben Abdellah, Fez 30070, Morocco

**Keywords:** pesticide biomonitoring, LC-MS/MS, farmers, toxicology, exposure risk factors, urinary biomonitoring

## Abstract

Pesticide exposure gradients between occupational, para-occupational, and general populations remain poorly characterized in North African agricultural contexts. This study evaluates urinary pesticide levels among farmers, indirectly exposed individuals, and a control group in Morocco’s Fez-Meknes region. A cross-sectional survey measured pesticide concentrations using LC-MS/MS in urine samples collected from 154 adults residing in both rural and urban areas. A questionnaire was used to gather information from participants regarding factors that may elevate the risk of pesticide exposure. The results revealed that farmers exhibited the highest concentrations of pesticides in their urine, including compounds classified as Ia/Ib by the World Health Organization. Indirectly exposed individuals showed moderate levels of contamination, with notable detections such as dichlofluanid (22.13 µg/L), while the control group had residual traces of neonicotinoids, notably imidacloprid (2.05 µg/L). Multivariate analyses revealed several sociodemographic factors significantly associated with increased pesticide exposure. The main risk factors identified included low education, residence in an agricultural area, and the consumption of untreated water (wells/rivers). Conversely, wearing personal protective equipment was associated with reduced urinary concentrations. This study highlights intense occupational exposure among farmers, secondary environmental contamination among residents living near treated areas, and the widespread dispersion of pesticide residues into urban areas.

## 1. Introduction

The intensification of environmental degradation, driven by accelerated urbanization and an expanding range of human activities, has resulted in the widespread dissemination of hazardous substances into natural ecosystems. As the use of synthetic chemicals has grown rapidly across industrial, agricultural, domestic, and technical domains, human populations are increasingly at risk of both direct and indirect exposure. This growing burden of chemical pollutants poses significant challenges to public and environmental health, especially in regions where surveillance, regulation, and risk mitigation strategies remain limited or inconsistently applied [1,2].

In contemporary agricultural systems, the use of pesticides presents a complex paradox. While contributing significantly to crop yield optimization and food availability, it simultaneously introduces substantial threats to both human health and environment [3]. From a chemical standpoint, pesticides are broadly grouped into several principal categories, including organochlorines, organophosphates, carbamates, pyrethroids, and pyrethrins. These compounds are primarily organic in nature and may be either synthetically produced or derived from botanical sources [4].

Global regulatory frameworks for pesticide management vary considerably. High-income countries typically enforce more rigorous legislation and oversight mechanisms. In contrast, many low- and middle-income nations face challenges related to insufficient institutional capacity, technical resources, and enforcement infrastructure, which limits the effective implementation of pesticide regulations [5].

Morocco’s extensive agricultural sector, spanning diverse agroecological zones, relies heavily on pesticide applications to enhance crop productivity and mitigate biotic stressors [6]. However, the escalating use of agrochemicals has raised critical public health concerns, particularly regarding occupational exposure among farming communities. Epidemiological evidence indicates inadequate adherence to safety protocols in rural areas, exacerbating exposure risks to pesticide residues [7].

Exposure to these chemicals can occur through inhalation, ingestion, or skin contact and is associated with various adverse effects [8]. Epidemiological studies have highlighted links between chronic exposure to pesticides and an increased risk of chronic kidney disease and neurodegenerative diseases, such as Parkinson’s disease and Alzheimer’s disease [9,10]. Additionally, some pesticides are classified as endocrine disruptors, interfering with the hormonal system and potentially causing reproductive problems, birth defects, and hormone-dependent cancers [11,12]. Children and pregnant women are particularly vulnerable to environmental and chemical exposures due to their developmental stages. Prenatal or early exposure to harmful substances can significantly impact neurological and cognitive development, leading to long-term consequences [13,14].

These impacts highlight the importance of biomonitoring as an essential tool, which not only allows for the precise quantification of individual and collective exposure by measuring residues of these substances in biological matrices such as blood, urine, or tissues [15,16] but also correlates this exposure to specific pathologies [17,18]. Biomonitoring plays a central role in identifying vulnerable populations, including children, pregnant women, and agricultural workers, due to their increased sensitivity or occupational exposure [19,20,21]. Furthermore, the data it provides constitute an essential scientific basis for the following: (i) strengthening pesticide regulations; (ii) promoting sustainable agricultural practices; (iii) developing prevention strategies adapted to at-risk groups [22].

The present article aims to investigate human exposure to a broad spectrum of pesticides through urinary biomonitoring in distinct population groups within the Fez-Meknes region of Morocco. By evaluating pesticide residue levels in different groups, this study offers novel insights into exposure gradients in a North African agricultural context. This work addresses a significant gap in the literature by providing relevant data from a region where biomonitoring studies remain scarce. The findings contribute to a better understanding of the risks of current agricultural practices and identify critical exposure items.

## 2. Materials and Methods

### 2.1. Chemicals and Reagents

All analytical standards (i.e., pesticides and metabolites), internal isotopic standards (IS), and solvents and reagents required for chromatographic analyses were purchased from certified suppliers (LGC standards, Teddington, United Kingdom, VWR International, Pennsylvania, the United States, Sigma-Aldrich, Darmstadt, Germany and Cluzeau Info Labo, Brive-la-Gaillarde, France). Purified water was obtained using a Millipore system, Darmstadt, Germany, and QuEChERS salts were supplied by Macherey Nagel, Düren, North Rhine-Westphalia, Germany. The complete list of analytical standards is provided in Supplementary File S1 for reference.

### 2.2. Study Area and Sampling Design

Two separate municipalities were selected as study sites in the Fez-Meknes region of Morocco: (1) the rural municipality of Aïn Aïcha, an agricultural area characterized by intensive pesticide use in cereal and olive crops; (2) the urban district of Fez, a primarily residential area with public green spaces.

These sites, among others in the Fez-Meknes region, had been previously and distinctively assessed for agricultural practices and environmental exposure levels by our research team [6]. We recruited 154 adult participants divided into three groups: 56 male farmers from Aïn Aïcha, 48 rural residents with indirect exposure (mainly women), and 50 urban controls (medical personnel from Fez). The observed sex distribution across the study groups reflects the socio-cultural and occupational roles specific to the rural Moroccan context. In this setting, farming activities involving direct handling and the application of pesticides are traditionally performed by men, which explains why the farmer group consists exclusively of males. Conversely, women in rural areas are more likely to be indirectly exposed through domestic tasks, occasional farm assistance, or environmental proximity to treated fields.

This study was conducted in a rural municipality with intensive agricultural activity and significant herbicide use, thus providing a relevant setting for assessing the health effects related to this exposure. Sample size calculation was performed using statistical power analysis (α = 0.05), taking into account the anticipated effect size, expected data variability, and the power required to detect significant associations between herbicide exposure and health parameters. Practical considerations related to participant accessibility, logistical constraints of recruitment and biological sample collection, and available resources were also incorporated in the final sample size determination. By ethical principles, all participants provided written informed consent after receiving full information on the objectives and conduct of the study. Data collection combined the following: (1) a standardized questionnaire administered face-to-face by trained interviewers, allowing the collection of sociodemographic characteristics, occupational history, and herbicide exposure patterns; (2) biological samples collected according to standardized protocols to ensure the quality of the analyses.

The farmers involved in the study were asked to report on the plant protection products they used. In our initial publication, we focused on glyphosate and its metabolite amino-methyl phosphonic acid (AMPA), given their widespread use in the region [13]. In this article, we extend our analysis to other identified pesticides. This complementary approach allows for a more comprehensive assessment of multiple exposures and their potential impacts on health and the environment in this agricultural area.

This study received ethical approval from the Regional Health Directorate of Fez-Meknes (ref. #91#/16/06/2023) and was conducted by the principles of the Declaration of Helsinki. Participants collected a first-morning urine sample in sterile 100 mL polypropylene containers. A minimum volume of 20 mL was required for a sample to be considered valid and included in the analysis.

A strict storage protocol was applied: samples were immediately refrigerated at 4 °C during transport to the ERES laboratory of the Faculty of Medicine, Pharmacy, and Odontology of Fez, and then stored at −80 °C for subsequent analysis within an average of 3 h post-collection. The assays were performed by the Pharmacology and Toxicology Laboratory of the Limoges University Hospital (France). After being stored at −80 °C for several weeks upon reception, the samples were thawed, and aliquots were prepared for further analysis. Aliquots intended for general screening were analyzed directly, while those designated for analysis of pyrethroid metabolites and dialkylphostates were stored at −80 °C until further testing implying that these specific aliquots underwent two freeze–thaw cycles.

To ensure data confidentiality, all personal information was anonymized before processing, by current regulations on the protection of personal data. This methodology ensured both the quality of the analyses and compliance with ethical standards throughout the study.

### 2.3. Pesticides Preparation and Extraction

#### 2.3.1. General Pesticide Screening

A liquid–solid extraction protocol was applied to 1 mL of a urine or serum sample, into which 15 µL of a 1:10 diluted working solution (Sol El LC) containing analytical standards (e.g., albendazole, carbendazim, phosalone) and an internal standard (IS) was spiked. The sample was then treated with 5 mL of acetonitrile (ACN) and 1 spatula of CHROMABOND^®^ QuEChERS Mix I (Macherey-Nagel, REF 730970.1, Sorbent-LOT 0125/30). This salt mixture consists of 4 g of anhydrous magnesium sulfate (MgSO_4_, ≥97%), 1 g of sodium chloride (NaCl), 0.5 g of disodium hydrogen citrate sesquihydrate (Na_2_H·citrate·1.5 H_2_O), and 1 g of trisodium citrate dihydrate (Na_3_·citrate·2 H_2_O). These salts facilitate phase separation and buffer the pH during extraction. After vortexing, the samples were centrifuged to induce phase separation. The upper organic layer (ACN) was carefully transferred to a new glass tube, followed by the addition of one extra spatula of MgSO_4_ to ensure the dehydration of residual aqueous content. A second vortex and centrifugation step were performed. The resulting supernatant was evaporated to dryness under a gentle nitrogen stream. The dried extract was reconstituted in 100 µL of a 50:50 mixture of methanol and aqueous buffer (MeOH/Tampon 8060), and 2 µL of the reconstituted sample were injected into a liquid chromatography system coupled to tandem mass spectrometry (LC-MS/MS). This protocol was validated across a range of concentrations (0.01 to 10 µg/L), with appropriate controls and fortified samples processed in parallel to ensure analytical performance.

#### 2.3.2. Pyrethroid Metabolites Biomonitoring

Quantification of urinary pyrethroid metabolites was performed after enzymatic hydrolysis with β-glucuronidase (from *Helix pomatia*), followed by liquid–liquid extraction with n-hexane and analysis by LC-MS/MS. The stable isotopes 3-PBA-^13^C and trans-Cl_2_CA-6D were used as IS.

A volume of 5 mL of urine was mixed with 1.25 mL of 1 M sodium acetate buffer (pH 4.8) and 25 µL of IS, and then 20 µL of β-glucuronidase was added. After incubation at 37 °C for 16 h, 6 mL of n-hexane and 1 mL of concentrated HCl (37%) were incorporated. The mixture was stirred for 10 min, and then centrifuged at 2000× *g* for 5 min. The organic phase was collected in a 15 mL glass tube, while the aqueous phase was extracted a second time with 6 mL of n-hexane under the same conditions. The two organic phases were combined, and then 3 mL of 0.1 M NaOH was added. After stirring (10 min) and centrifugation (2000× *g*, 5 min), the upper phase was removed. Then, 6 mL of n-hexane and 200 µL of HCl (37%) were added to the residue. After further stirring and centrifugation, the organic phase was collected and evaporated to dryness under a nitrogen stream.

The dry residue was reconstituted in 80 µL of a water/0.1% formic acid mixture (70:30, *v*/*v*), and 10 µL of this solution was injected into the LC-MS/MS system. Calibration curves were established by applying the same procedure to standards spiked with the appropriate working solutions.

#### 2.3.3. Organophosphate Metabolite Biomonitoring

An analysis of urinary metabolites of organophosphorus pesticides was performed by LC-MS/MS after an organic solvent extraction step. Specific stable isotopes were used as ISs for determination.

The assay was conducted as follows: 20 µL of IS was added to 2 mL of urine sample. Then, 1 mL of 6 M HCl, 5 mL of diethyl ether, and 4 g of sodium chloride were added. The mixture was stirred for 15 min and centrifuged for 5 min at 2000× *g*. The organic phase was recovered into a 15 mL glass tube, while the remaining aqueous phase was re-extracted with 5 mL of ethyl acetate, stirred, and centrifuged under the same conditions as previously described. The two organic phases were combined, and then evaporated to dryness under nitrogen flow. The resuspension of the dried samples was performed in 1 mL of (50/50) 2 mM ammonium formate (pH 3) and methanol, and finally, 5 µL of the solution was injected into the LC-MS/MS system. The calibration standards were subjected to the same treatment after they were spiked with the appropriate volume of the working solutions.

### 2.4. Instrumental Analysis

#### 2.4.1. General Pesticide Screening

The LC-MS/MS system used in this assay is composed of an LC-MS 8060 triple quadrupole mass spectrometer and a Shimadzu NEXERA X2 series, Kyoto, Japan. The column used for the separation is a Raptor Biphenyl column (100 × 2.10 mm, 2.7 µm particles), Pennsylvania, United States.

The mobile phases for the pumps were prepared as follows: for pump A, a combination of 0.002% formic acid and ammonium formate (2 mmol/L) in water was used, while for pump B, the same combination was used but this time in methanol instead of water. The flow rate was set at 0.3 mL/min and phase B was set at 10% from 0 to 0.5 min, 60% from 13 to 28 min, and 100% until the end of the run at 35 min.

The positive and negative ionization modes were used in the identification and quantification of pesticides. Pesticide identification and quantification were carried out in both positive and negative ionization modes using multiple reaction monitoring (MRM), targeting one quantifier and one qualifier ion for each of the 320 compounds included in this targeted screening [23]. For a positive identification, the ratio between the quantifier and qualifier ion transitions had to be within ±20% of the ratio determined from the calibration standards.

#### 2.4.2. Pyrethroid Metabolites

The LC-MS/MS system used in this assay is composed of an AB SCIEX API 5500 QTrap triple quadrupole mass spectrometer and a Shimadzu LC-20AD. The column used for the separation is an Atlantis T3 column (150 × 2.10 mm, 5 µm particles) (Waters, Milford, MA, USA). The mobile phases for the pumps were prepared as follows: phase A contained 0.1% formic acid and phase B was a mixture of (95/5) methanol acidified with 0.1% formic acid. For phase A, the flow rate was set at 0.2 mL/min with a gradient of phase B that increases from 50% to 80% between 0.5 and 10 min and then decreases to 50% at 12 min until the end of run at 18 min. The identification and quantification of 3-PBA, 4-FPBA, Cl_2_CA (cis and trans), and Br_2_CA were conducted in negative ionization mode using MRM. For each compound, a quantifier ion transition was used (213.0/92.9 for 3-PBA, 231.0/93.1 for 4-FPBA, 208.9/36.9 for Cl_2_CA, and 342.9/80.8 for Br_2_CA), along with a corresponding qualifier ion transition (213.0/65.1, 231.0/65.1, 207.0/35.0, and 296.8/80.9, respectively). To confirm identification, the ratio between the quantifier and qualifier transitions, originating from the same precursor ion, was required to fall within ±20% of the ratio established by calibration standards

#### 2.4.3. Organophosphate Metabolite

Analyses were performed using an LC-MS/MS system integrating a triple quadrupole mass spectrometer (Shimadzu 8060) coupled with the NEXERA X2 chromatographic system (Shimadzu, Japan). Chromatographic separation was performed on an INERTSIL ODS-3 column (100 × 2.1 mm, 5 µm, GL Sciences Inc., Tokyo, Japan). Mobile phase A consisted of a 2 mM ammonium formate solution (pH 3), while phase B consisted of a 2 mM acetonitrile/ammonium formate mixture (90:10, *v*/*v*; pH 3). The gradient program included an isocratic elution at 90% B from 0 to 3 min, followed by a reduction to 20% B between 3 and 7 min, and then a return to 90% B for up to 9 min. The mobile phase flow rate was maintained at 0.2 mL/min. The identification and quantification of the metabolites DMP, DMTP, DMDTP, DEP, DETP, and DEDTP were performed in negative MRM mode, following specific transitions for each compound (quantifier and qualifier ions).

The LC-MS/MS method was validated according to standard analytical criteria, including linearity, correlation coefficient, limits of detection (LOD) and quantification, precision, accuracy, and extraction yield. This method has been accredited by COFRAC.

### 2.5. Statistical Analysis

Descriptive statistics, including frequency tables, were performed using IBM SPSS Statistics 26.0 software (IBM Corp., Armonk, NY, USA, 2019) to present demographic characteristics and pesticide exposure levels. All statistical tests were performed with a significance level of α = 0.05. Pesticide concentrations are expressed in µg/L. Samples with concentrations below the LOD were assigned a zero value for statistical analysis. This conservative approach minimizes exposure overestimation in population-level comparisons. Principal component analysis, partial least squares-discriminant analysis (PLS-DA), and multiple regression were performed using the R exploratory multivariate data analysis ropls package version 1.26.428. A map showing the location of the study area was prepared using ArcGIS 10.3.1 software. Figures were generated using GraphPad Prism (version 9.0) and Adobe Illustrator 2020 for graphical refinement.

## 3. Results

### 3.1. Survey Data

**Figure 1:**
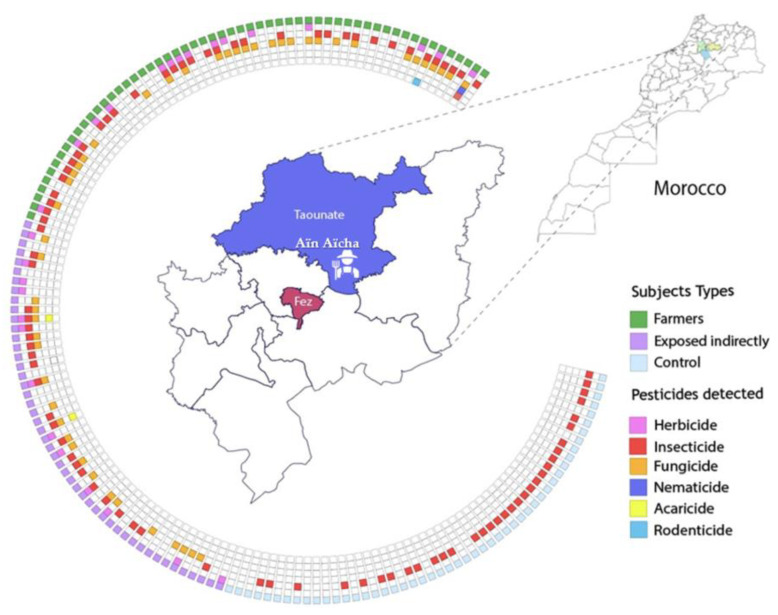
Classification of participants (N = 154) according to the categories of pesticides detected: representation by concentric rings showing (1) the exposure groups (farmers, indirectly exposed, controls) in the outer ring, and (2) the types of contaminants (herbicides, insecticides, fungicides, nematicides, acaricides, rodenticides) in the inner rings.

Table 1 presents the sociodemographic characteristics of the 154 participants from the Fez-Meknes region of Morocco, divided into three groups: farmers (56 individuals), indirectly exposed individuals (48 individuals), and the control group (50 individuals). (Figure 1).

The mean age of participants was 47.45 ± 14.48 years, with significant differences between groups (*p* < 0.05): farmers were on average older (53.53 ± 13.88 years) than indirectly exposed individuals (49.83 ± 14.38 years) and the control group (38.36 ± 10.39 years).

Farmers were exclusively male (100%), while the indirectly exposed and control groups were 87.5% and 78% female, respectively (*p* < 0.001). In terms of education, most farmers had no education (51.7%) or had only attended primary school (44.6%), whereas the control group had a higher level of education, with 28% having reached secondary school and 72% having reached university level (*p* < 0.001). Regarding place of residence, all farmers lived on farms, compared to 87.5% of indirectly exposed individuals and no individual in the control group (*p* < 0.001). The source of drinking water also varied significantly between groups: 87.5% of farmers and 93.7% of indirectly exposed individuals used wells or rivers, while 100% of the control group used tap water (*p* < 0.001) (Table 1). Among the participants, 75% of farmers consumed mainly farm products while all controls reported consuming mainly market products (*p* < 0.001). While all farmers use pesticides, only 23.2% of them (*n* = 13) adopt personal protective equipment (PPE).

### 3.2. Pesticide Contamination in Urine Samples

A total of 30 unique pesticide compounds were detected (above the LOD) in samples collected from 56 farmers, 48 indirectly exposed individuals, and 50 control individuals. Table 2 shows the results of the detection of these 30 pesticides in the three groups studied: farmers, indirectly exposed individuals, and the control group.

**Figure 2:**
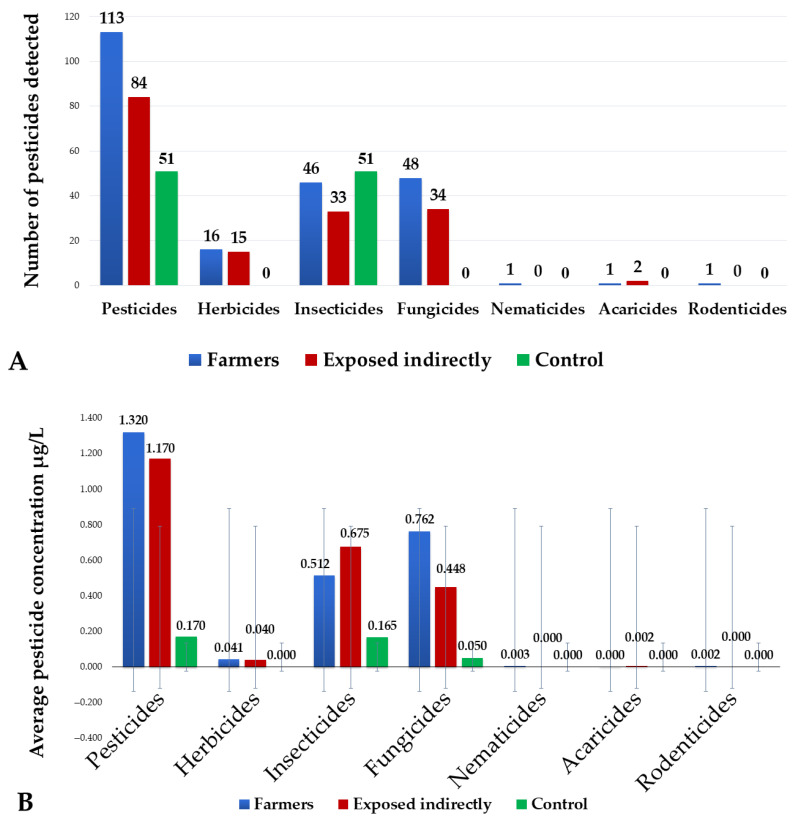
Histogram illustrating the number of pesticides detected (**A**) and the average pesticide concentration µg/L (**B**) in the three groups studied: farmers (blue), indirectly exposed individuals (red), and control group (green).

These pesticides are distributed as follows: 10 insecticide ingredients, 11 fungicide ingredients, 5 herbicide ingredients, 2 acaricides, 1 nematicide, and 1 rodenticide. They were classified according to their hazard by the World Health Organization (WHO). Farmers have the highest exposure, with frequent detections of thiamethoxam (24 detections), dichlofluanid (10 detections), and trichlorophenol 2,4,6 (12 detections). Maximum concentrations were observed for pthalimide (30.90 µg/L), endosulfan-lactone (8.24 µg/L), and tetrachlorvinphos (7.36 µg/L). This was particularly the case for dodine (15 detections) and thiamethoxam (15 detections). Indirectly exposed individuals also showed significant levels of exposure, characterized by high levels of dodine (31.3% of samples) and thiamethoxam, as well as notable maximum concentrations of dichlofluanid (22.13 µg/L) and dinotefuran (18.62 µg/L). In addition, the control group revealed significant contamination, particularly by imidacloprid (28 detections) and thiamethoxam (14 detections), with concentrations reaching 2.05 µg/L and 0.34 µg/L, respectively. It is important to note that the number of unique pesticide types (*n* = 30) differs from the total number of individual detections, as several pesticides were found in multiple participants. The diagram shows the distribution and mean concentrations of pesticides (in µg/L) detected in the three groups (Figure 2).

**Figure 3:**
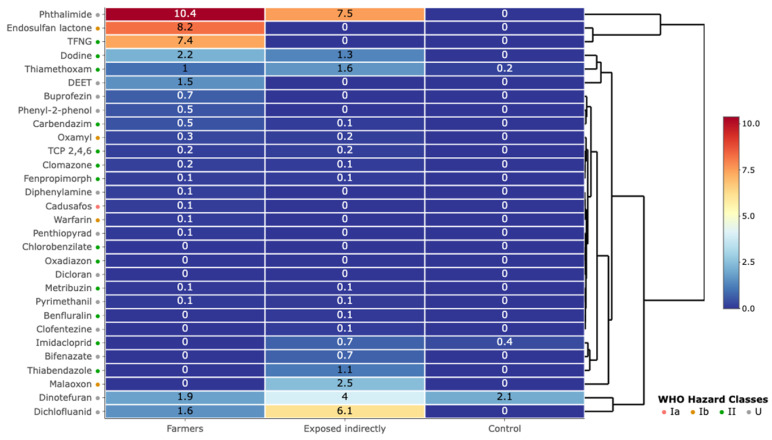
Supervised heat map representing the average concentrations of the 30 detected contaminants (rows) in farmers (N = 56), indirectly exposed individuals (N = 48) and the control group (N = 50), divided into three classes (columns). The colored dots indicate the pesticide hazard classes according to the WHO classification: class Ia (red), class Ib (orange), class II (green), and class U (gray).

Farmers had the highest number of pesticides detected (113), with a predominance of insecticides (46) and fungicides (48). The highest mean concentrations were observed for insecticides (1.24 ± 0.18 µg/L) and fungicides (0.83 ± 0.12 µg/L) in this group. Indirectly exposed individuals showed intermediate levels, with a 40–60% reduction in concentrations compared to farmers. The control group mainly showed residual traces. Other pesticide categories, including herbicides, acaricides, nematicides, and rodenticides, were detected at frequencies below 15% and at concentrations not exceeding 0.2 µg/L in all groups studied. In farmers, the highest concentrations were observed for several compounds, with particularly notable values for phthalimide (10.36 µg/L), endosulfan lactone (8.24 µg/L), and tetrachlorvinphos (7.36 µg/L). Indirectly exposed individuals had significant levels of phthalimide (7.5 µg/L), dichlofluanid (6.13 µg/L), and dinotefuran (4 µg/L), while the control group mainly had residual contamination with neonicotinoids (dinotefuran at 2.1 µg/L and imidacloprid at 0.4 µg/L) (Figure 3).

Pesticides classified Ia/Ib by the WHO (cadusafos, endosulfan lactone, oxamyl) were exclusively detected in farmers, with concentrations reaching 8.24 µg/L for endosulfan lactone. In addition, certain compounds such as dichlofluanid and phthalimide, although classified as U, present particularly high concentrations (>5 µg/L).

**Figure 4:**
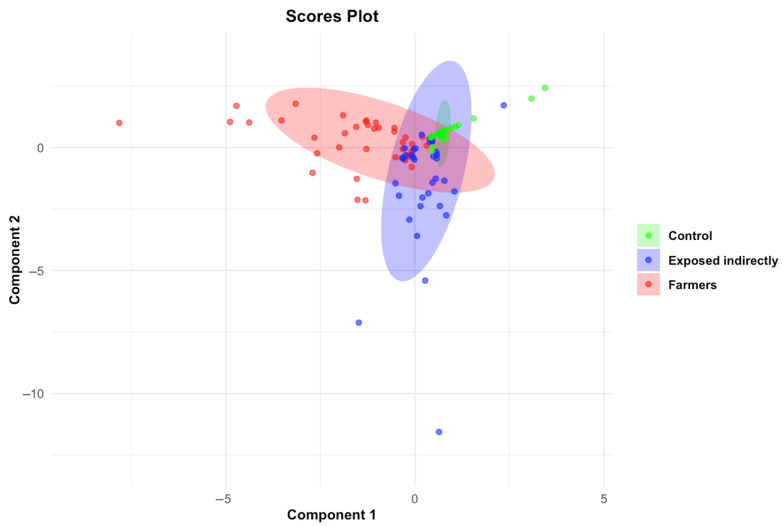
PLS-DA score plot based on the 30 detected contaminants, showing the separation between the three groups: farmers (N = 56; red), indirectly exposed individuals (N = 48; blue), and control group (N = 50; light green).

PLS-DA score analysis revealed a clear separation between the three study groups (farmers, indirectly exposed individuals, and control group) based on urinary pesticide concentrations (Figure 4).

**Figure 5:**
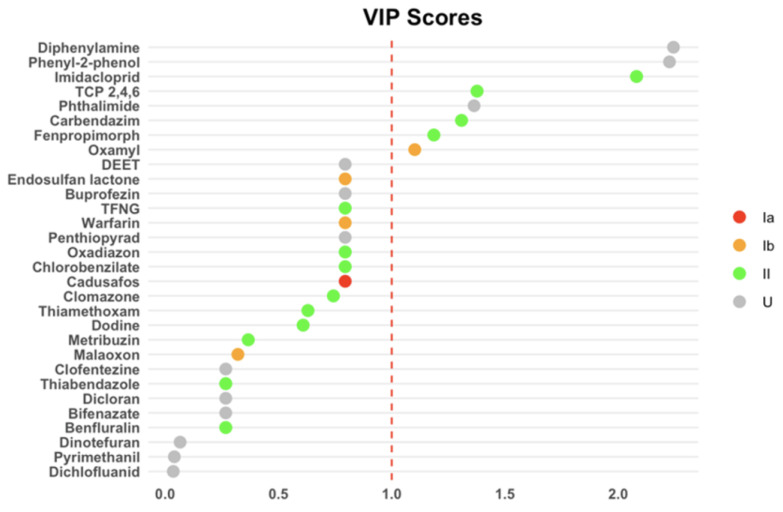
Dot plot displaying VIP scores for the most important pesticides identified by the PLS-DA model. The colored dots indicate the pesticide hazard classes according to the WHO classification: class Ia (red), class Ib (orange), class II (green), and class U (gray).

Farmers clustered in a distinct region of the factorial design, reflecting specific pesticide concentration profiles. The control group clustered in an opposite area, indicating significantly lower exposure levels. Indirectly exposed individuals occupied an intermediate position, suggesting moderate exposure. VIP analysis identified eight pesticides as main discriminant markers (VIP > 1) between exposure groups: diphenylamine, 2-phenylphenol, imidacloprid, TCP 2,4,6, phthalimide, carbendazim, fenpropimorph, and oxamyl (Figure 5).

**Figure 6:**
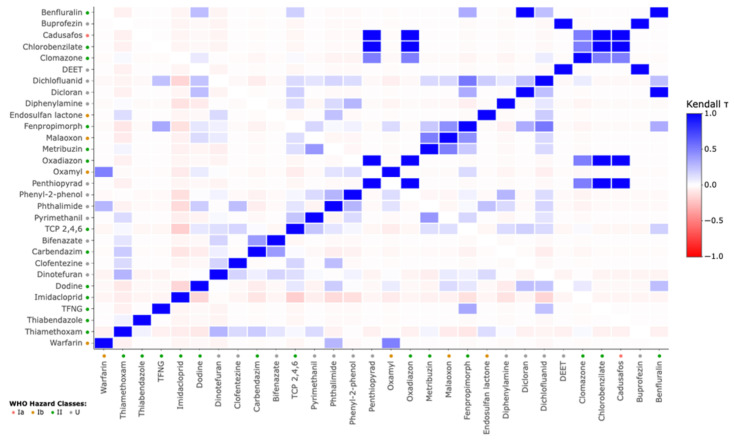
Sorted correlation matrix of the 30 contaminants detected in urine samples. Kendall’s τ coefficients are used to represent the strength and significance of the correlations, according to the scale shown in the legend to the right. The colored dots indicate the pesticide hazard classes according to the WHO classification: class Ia (red), class Ib (orange), class II (green), and class U (gray).

Correlation matrix analysis revealed distinct patterns of pesticide co-exposure. Strong correlations (τ > 0.6) were observed between WHO class Ia/Ib insecticides (cadusafos, endosulfan lactone) and commonly used neonicotinoids (imidacloprid, thiamethoxam). Triazole fungicides, such as dodine and carbendazim, showed intermediate-to-strong correlations (τ = 0.58–0.65) and formed a distinct cluster (Figure 6).

### 3.3. Correlation of Urinary Pesticide Concentrations with Sociodemographic Factors

Multivariate regression analysis, the detailed results of which are presented in Table 3, assessed the associations between urinary concentrations of different types of pesticides and various sociodemographic and behavioral variables. It was found that there was no statistically significant association between the age or sex of the participants and the urinary concentrations of the pesticides studied. However, this analysis revealed several significant associations. More specifically, individuals with a university education level had significantly lower urinary concentrations of acaricides (*p* = 0.014) compared to those without formal education. Furthermore, residence in an agricultural environment was associated with higher urinary concentrations of acaricides (*p* = 0.001). Similarly, the consumption of water from wells or rivers was linked to higher urinary concentrations of rodenticides. Finally, the use of Personal Protective Equipment (PPE) was associated with significantly lower urinary concentrations of herbicides (*p* = 0.019) and rodenticides (*p* = 0.008). These four variables retained after the multivariate analysis were then included in a supervised multivariate approach to better elucidate their individual contributions to pesticide contamination.

## 4. Discussion

In this research, an exploratory observational survey was conducted to assess pesticide exposure among residents of the Fez-Meknes region. This work is a continuation of an initial investigation on urinary biomonitoring of glyphosate and its main metabolite, AMPA, among farmers, indirectly exposed individuals, and a control group [13]. Although this initial study demonstrated exposure to these substances, available data on other pesticide classes remain limited. Therefore, the present study aims to expand knowledge by examining exposure to a broader range of pesticides from various chemical families to obtain a more comprehensive assessment of toxicological risk for this population. We measured pesticide concentrations in urine samples from 154 adults living in rural and urban areas, using validated analytical methods [23]. A control was evaluated at an interval of 10 samples at varying levels, varying between the LOQ and a median concentration level. All aspects related to analytical validation, including instrumental calibration and quality assurance procedures, quality control using replicates, and the assessment of matrix effects and sensitivity, are comprehensively detailed in Supplementary File S2.

Urine constitutes a primary biological matrix for biomonitoring due to its ability to reflect recent exposure levels, making it a valuable tool for assessing occupational and environmental risks [24]. Urine collection is non-invasive and can be easily conducted in large population studies, facilitating extensive biomonitoring efforts [25]. We therefore assessed pesticide exposure among farmers, their indirectly exposed wives, and the control group.

Our results indicate heterogeneous but widespread pesticide contamination across all groups studied, with a marked prevalence among farmers, reflecting acute occupational exposure. A total of 30 active substances were detected above the LOD, including a majority being insecticides (10 compounds) and fungicides (11 compounds), reflecting their dominant use in regional phytosanitary practices [6]. The exposure burden is significantly higher among farmers, both in terms of frequency and concentration, characterized by the detection of 113 pesticides including 46 insecticides and 48 fungicides, directly reflecting intensive agricultural practices. The presence at particularly high concentrations of phthalimide (30.90 µg/L) and endosulfan-lactone (8.24 µg/L), two compounds recognized as endocrine disruptors (EDCs), raises major public health concerns due to their demonstrated ability to interfere with hormonal regulatory mechanisms. These substances exert their harmful effects mainly by binding to hormone receptors, disrupting the synthesis, metabolism and activity of endogenous hormones, including estrogens and androgens, essential for the maintenance of many functions [26,27,28]. This biochemical interaction can lead to significant hormonal imbalance, which can compromise human health systemically. Indeed, chronic exposure to these EDCs is increasingly correlated with an increased incidence of serious pathologies such as hormone-dependent cancers (especially breast and prostate cancers), metabolic disorders including obesity and type 2 diabetes, as well as various alterations of reproductive function, such as infertility and abnormalities of embryonic and fetal development [29,30].

The exclusive detection of WHO Ia/Ib pesticides, including cadusafos and oxamyl, in the agricultural population represents a critical public health problem. Chronic exposure to these substances can lead to various neurological disorders, hence the need to raise awareness among agricultural populations and take preventive measures [31]. The classification of dichlofluanid and phthalimide as “low hazard” (U) despite their high concentrations (>5 µg/L) raises serious concerns regarding traditional toxicological assessments. These classifications often neglect chronic effects at low doses and the possibility of combined effects, which may lead to an underestimation of the risks associated with these substances. A recent ecotoxicological study demonstrates that dichlofluanid has significant chronic toxicity in marine organisms, with adverse effects observed at environmentally relevant concentrations as low as 1 µg/L in sea urchin embryos and 100 µg/L in copepods [32]. Chronic exposure to low doses can lead to cumulative health effects, as evidenced by studies linking pesticide exposure to chronic respiratory diseases [33].

These findings call for a rethinking of conventional toxicological frameworks, including incorporating the potential effects of repeated exposures. Our correlation matrix (Figure 6) highlights co-detections in pairwise combinations of neonicotinoids, triazole fungicides, and organophosphate insecticides belonging to different chemical classes. This pattern indicates potential cumulative or synergistic effects via overlapping biological mechanisms such as endocrine disruption and neurotoxicity. A recent study has proven that co-exposure to pyrethroids and phenylpyrazoles results in synergistic neurotoxicity via shared mitochondrial signaling and proteolytic chimneys [34].

In this context, approaches such as cumulative risk assessment (e.g., EPA Cumulative Risk Index) or Adverse Outcome Pathways offer promising integrative frameworks that are better suited to the complex exposures observed in agricultural and peri-agricultural populations [35].

In indirectly exposed individuals, the analysis reveals an intermediate exposure profile, with an estimated 40–60% decrease in concentrations compared to farmers. However, the frequent detection of pesticides such as dodine (31.3% of samples) and thiamethoxam, as well as the high concentrations measured for dichlofluanid (22.13 µg/L) and dinotefuran (18.62 µg/L), indicate diffuse environmental contamination, probably due to air drift, dust transport or soil leaching [36,37].

On the other hand, the detection of imidacloprid residues (28 samples, up to 2.05 µg/L) and thiamethoxam (14 samples) in the control group confirms the environmental dispersion of neonicotinoids in Morocco. Although these substances are subject to restrictions in the European Union, their use remains widespread in Moroccan agriculture, particularly in intensive crops (market gardening, citrus fruits, and cereals) [38]. These compounds persist in the environment and exhibit high mobility, with a demonstrated ability to diffuse into aquatic ecosystems and accumulate in food chains [39]. Their detection, even at low doses, remains a concern due to their potential neurotoxic effects, particularly on brain development [40,41].

Multivariate regression analysis (Table 3) highlighted key determinants of pesticide exposure in our study population, highlighting the complexity of exposure pathways. We observed that individuals with a higher level of education, particularly university education, had significantly lower urinary acaricide concentrations (*p* = 0.014). Similar observations were reported in an integrated analysis of three epidemiological studies: the PIPAH cohort in the UK, which focused on professional pesticide users, a study of farmers in Malaysia, and the PESTROP project in Uganda [42]. Higher education levels are generally associated with a better understanding of the risks associated with pesticide use, an increased awareness of potential health effects, and more frequent adoption of personal protective measures [43]. In Morocco, it has been shown that the majority of farmers in the Fez-Meknes region have a low level of education, which constitutes an obstacle to the adoption of safe agricultural practices [6].

Moreover, residence in an agricultural setting is frequently correlated with increased pesticide exposure, due to proximity to application sources [44,45]. A statistically significant association was observed between place of residence and urinary concentrations of acaricides (*p* = 0.001), confirming persistent passive environmental exposure in individuals living in these areas. This exposure can occur through inhalation of contaminated particles in the air, skin contact with household surfaces or ingestion of contaminated dust, especially in homes close to treated fields [46,47]. In this context, the quality of water used for domestic purposes could represent an additional source of exposure. Indeed, the consumption of water from untreated wells or rivers has been correlated with significantly higher urinary concentrations of rodenticides. This result suggests possible contamination of water resources by agricultural residues, via runoff, infiltration, or drift of products applied to crops [48]. Thus, the combination of several environmental vectors, such as air, dust, surfaces, and water, could explain the increased levels of biologically detectable pesticides in human biological matrices in rural areas, even in the absence of direct occupational exposure [49,50].

Along with passive exposure experienced by rural residents, active or occupational exposure represents another major route of pesticide absorption, particularly among farmers [51]. In this context, the use of PPE such as gloves, masks, suits, or goggles appears to be an effective preventive measure [52]. Our results show that regular PPE use was associated with significantly lower urinary concentrations of herbicides and rodenticides, consistent with recent research findings showing that behavioral practices, including PPE use, can alter biological exposure profiles [53,54]. For example, a longitudinal study embedded in the ECHO program in the United States found that changes in behavior were associated with measurable changes in urinary concentrations of various contaminants, including pesticides [55]. However, in many agricultural areas, the use of PPE remains insufficient or inappropriate, often due to economic barriers, a lack of awareness of the risks or the perception that such equipment hinders comfort and efficiency at work [56,57,58].

This study provides a comprehensive assessment of pesticide exposure in Morocco using a biomonitoring approach, presenting several major strengths. First, the inclusion of three distinct groups (farmers, indirectly exposed individuals, and urban controls) allows for robust comparisons of occupational and environmental exposure profiles. The use of COFRAC-accredited LC-MS/MS methods ensures high analytical reliability, with the detection of 30 pesticide compounds belonging to different classes. The study design integrates both quantitative exposure data and detailed sociodemographic information, allowing for a nuanced analysis of risk factors. Moreover, the application of PLS-DA multivariate analysis effectively differentiates exposure profiles between groups.

However, several methodological limitations must be acknowledged regarding the assessment of long-term exposure trends or the establishment of direct causal relationships between pesticide exposure and potential health outcomes. Although urinary biomonitoring is a valuable and validated tool for occupational and environmental risk assessment, offering a non-invasive and easily implementable method for population studies, it primarily reflects recent exposure. Therefore, the urine analyses conducted here cannot reflect chronic accumulation or seasonal variations in pesticide use.

In our study, we performed a one-time spot urine sampling, which provides a snapshot of recent exposure rather than a long-term exposure estimate. The majority of the pesticides detected in our cohort are characterized by short biological half-lives, typically ranging from a few hours to a few days. For example, thiamethoxam t_1_/_2_ is 1–3 h in human plasma, with rapid urinary excretion of its metabolite clothianidin [59]; imidacloprid, t_1_/_2_ ≈ 5 h in humans, with urinary elimination of hydroxylated and nitroso metabolites; carbendazim, t_1_/_2_ ≈ 6–12 h in humans; rapid first-pass metabolism [60]; phthalimide (a metabolite of folpet and captan), rapidly hydrolyzed and cleared within 24 h in rodent models [61]. Given these short half-lives, the detection of parent compounds or metabolites in urine indicates recent exposure, typically within the preceding 24–72 h. However, without longitudinal sampling or dietary/exposure diaries, it is not possible to extrapolate this to average or chronic exposure levels.

Furthermore, the targeted selection of the municipalities of Aïn Aïcha and Fez, while relevant for our local objective, limits the generalizability of the results to all Moroccan agricultural areas. Regarding the demographic characteristics of the sample, the marked gender imbalance (100% male farmers, 87.5% female indirectly exposed individuals, and 78% female controls) reflects the socio-professional reality on the ground in this region of Morocco. Although this may a priori raise questions about the interpretation of sex-specific exposure patterns, our multiple regression model included sex as an explanatory variable, and the results indicated a lack of statistically significant association between participant sex and urinary concentrations of the studied pesticides for the compounds considered. Other methodological limitations include the failure to account for potential pesticide residues in consumed food, as well as in air, soil, and surface water. Thus, although urinary biomonitoring reflects overall exposure, precise identification of exposure pathways, particularly dietary and environmental, remains limited. Future research should include these measurements to better characterize exposure sources.

## 5. Conclusions

Our research highlights significantly different pesticide exposure levels according to the proximity to agricultural activities in the Fez-Meknes region. Farmers exhibit the highest concentrations, including pesticides classified as highly hazardous by the WHO. Intermediate levels of contamination are observed among those indirectly exposed, and residual traces are detected even in urban areas.

The study reveals significant associations between elevated pesticide levels and various sociodemographic factors, including lower educational attainment, residence in agricultural areas, and the use of untreated water sources. In contrast, wearing PPE is associated with a significant reduction in exposure risk. These findings underscore the urgent need for strengthened pesticide management policies and targeted educational interventions, particularly in rural settings, to reduce health risks linked to pesticide exposure and promote safer agricultural practices.

## Figures and Tables

**Table 1:** Demographic characteristics of the total study population.

Feature	Overall	Farmers	Exposed Indirectly	Control	*p* Value
Cohort	154	56	48	50	
Age (years)					0.232 ^a^
Mean ± SD	47.45 ± 14.48	53.53 ± 13.88	49.83 ± 14.38	38.36 ± 10.39	
Median	47.00	53.00	50.50	34.50	
Range	[22–87]	[29–80]	[22–87]	[26–70]	
Interquartile range	23.50	21.00	21.50	14.75	
Gender					<0.001 ^b^
Man	73 (47.4%)	56 (100%)	6 (12.5%)	11 (22%)	
Female	81 (52.6%)	0 (0%)	42 (87.5%)	39 (78%)	
Education					<0.001 ^c^
None	60 (38.96%)	29 (51.7%)	31 (64.6%)	0 (0%)	
Primary school	40 (25.97%)	25 (44.6%)	15 (31.25%)	0 (0%)	
Secondary school	18 (11.68%)	2 (3.7%)	2 (4.15%)	14 (28%)	
University	36 (23.39%)	0 (0%)	0 (0%)	36 (72%)	
Living on farm					<0.001 ^b^
Yes	98 (63.64%)	56 (100%)	42 (87.5%)	0 (0%)	
No	56 (36.36%)	0 (0%)	6 (12.5%)	50 (100%)	
Drinking water source					<0.001 ^b^
Tap	64 (41.56%)	7 (12.5%)	3 (6.25%)	50 (100%)	
Wells and rivers	90 (58.44)	49 (87.5%)	45 (93.75%)	0 (0%)	
Origin of the food consumed					<0.001 ^b^
Market	86 (55.9)	14 (25)	22 (45.8)	50 (100)	
Farm	68 (44.1)	42 (75)	26 (54.2)	0 (0)	
Pesticide use					0.005 ^b^
Yes	80 (51.94%)	56 (100%)	24 (50%)	0 (0%)	
No	74 (48.06%)	0 (0%)	24 (50%)	50 (100%)	
Use of PPE					<0.001 ^b^
Yes	13 (8.5)	13 (23.2)	0 (0)	0 (0)	
No	141 (91.5)	43 (76.8)	48 (100)	50 (100)	

^a^ Student *t*-test; ^b^ Chi square test; ^c^ Fisher’s exact test. Percentages in parentheses represent proportions within each category for each group (farmers, exposed indirectly, controls). PPE, personal protective equipment.

**Table 2:** Urinary pesticide contaminant profiles in 154 adults.

Pesticide			>LOD			Maximum Concentration		
Substance	Type	Hazardous Class	Farmers	Exposed Indirectly	C	Farmers	Exposed Indirectly	C
Benfluralin	Herbicide	II	0	1	0	0.00	0.05	0
Buprofezin	Insecticide	U	1	0	0	0.66	0	0
Cadusafos	Nematicide	Ia	1	0	0	0.14	0	0
Chlorobenzilate	Acaricide	II	1	0	0	0.02	0	0
Clomazone	Herbicide	II	2	2	0	0.18	0.11	0
DEET	Insecticide	U	1	0	0	1.54	0	0
Dichlofluanid	Fungicide	U	10	7	0	7.10	22.13	0
Dicloran	Fungicide	U	0	1	0	0.00	0.09	0
Diphenylamine	Fungicide	U	8	0	0	0.23	0	0
Endosulfan Lactone	Insecticide	Ib	1	0	0	8.24	0	0
Fenpropimorph	Fungicide	II	5	4	0	0.18	0.16	0
Malaoxon	Insecticide	Ib	0	2	0	0.00	3.9	0
Metribuzin	Herbicide	II	1	1	0	0.05	0.052	0
Oxadiazon	Herbicide	II	1	0	0	0.02	0	0
Oxamyl	Insecticide	Ib	3	1	0	0.50	0.15	0
Penthiopyrad	Fungicide	U	1	0	0	0.06	0	0
Phenyl 2, phenol	Fungicide	U	10	0	0	1.21	0	0
Phthalimide	Fungicide	U	9	4	0	30.90	20.40	0
Pyrimethanil	Fungicide	U	1	2	0	0.06	0.10	0
Trichlorophenol 2,4,6	Herbicide	II	12	12	0	0.29	0.34	0
Bifenazate	Acaricide	U	0	1	0	0.00	0.73	0
Carbendazim	Fungicide	II	4	2	0	0.92	0.15	0
Clofentezine	Acaricide	U	0	1	0	0.00	0.11	0
Dinotefuran	Insecticide	U	16	13	9	4.82	18.62	4.44
Dodine	Fungicide	II	1	15	0	2.20	2.97	0
Imidacloprid	Insecticide	II	0	2	28	0.00	1.29	2.05
Tetrachlorvinphos	Insecticide	II	1	0	0	7.36	0	0
Thiabendazole	Fungicide	II	0	1	0	0.00	1.14	0
Thiamethoxam	Insecticide	II	24	15	14	9.26	9.31	0.34
Warfarin	Rodenticide	Ib	1	0	0	0.11	0	0

Concentration distribution by WHO Hazard is defined according to the WHO recommended classification of pesticides by hazard, including the following: class Ia, extremely hazardous; class Ib, highly hazardous; class II, moderately hazardous; U, unlikely to present acute hazard. LOD, limit of detection; C, control.

**Table 3:** Summary of the multiple regression model: concentrations by pesticide type vs. explanatory variables.

Var	Herbicide (μg/L)			Insecticide (μg/L)			Fungicide (μg/L)			Acaricide (μg/L)			Nematicide (μg/L)			Rodenticide (μg/L)		
	M	Est Coeff.	*p*	M	Est Coeff.	*p*	M	Est Coeff.	*p*	M	Est Coeff.	*p*	M	Est Coeff.	*p*	M	Est Coeff.	*p*
Age	-	0.001 (−0.001–0.001)	0.645	-	−0.008 (−0.022–0.007)	0.297	-	−0.088 (−0.031–0.012)	0.390	-	−0.101 (-)	0.313	-	−0.076 (-)	0.443	-	−0.151 (-)	0.150
Gender		−0.007 (−0.032–0.017)	0.562		−0.125 (−0.542–0.293)	0.556		−0.439 (−1.048–0.171)	0.157		0.029 (−0.003–0.004)	0.765		0.009 (−0.004–0.004)	0.926		−0.099 (−0.005–0.002)	0.337
Man	0.034	-		0.444	-		0.675	-		0.000	-		0.001	-		0.001	-	
Female	0.021			0.457			0.183			0.001			0.000			0.000		
Education		−0.014 (−0.031–0.004)	0.134		−0.255 (−0.558–0.047)	0.097		−0.353 (−0.794–0.089)	0.117		−0.419 (−0.006–−0.001)	0.014		−0.042 (−0.003–0.003)	00.805		−0.223 (−0.004–0.001)	0.209
None	0.044	-		0.637	-		0.821	-		0.001	-		0.000	-		0.001	-	
Primary school	0.035			0.510			0.324			0.000			0.003			0.000		
Secondary school	0.007			0.271			0.078			0.000			0.000			0.000		
University	0.000			0.161			0			0.000			0.000			0.000		
Living on farm	-	−0.018 (−0.062–0.026)	0.419	-	0.085 (−0.564–0.938)	0.623	-	−0.133 (−1.229–0.962)	0.810	-	0.566 (0.004–0.017)	0.001	-	−0.214 (−0.013–0.003)	0.208	-	0.083 (−0.005–0.008)	0.639
Yes	0.040	-		0.530	-		0.654	-		0.000	-		0.001	-		0.001	-	
No	0.004			0.313			0.000			0.002			0.000			0.000		
Drinking water	-	−0.009 (−0.046–0.028)	0.644	-	0.247 (−0.093–1.168)	0.094	-	−0.298 (−1.219–0.623)	0.524	-	0.110 (−0.003–0.007)	0.445	-	−0.369 (−0.015–−0.002)	0.011	-	−0.072 (−0.007–0.004)	0.634
Tap	0.008	-		0.186	-		0.782	-		0.000	-		0.002	-		0.000	-	
Wells and rivers	0.039			0.621			0.001			0.001			0.000			0.001		
Origin of the food consumed	-	0.009 (−0.017–0.034)	0.494	-	0.066 (−0.291–0.571)	0.521	-	−0.048 (−0.677–0.582)	0.881	-	−0.195 (−0.007–0.000)	0.054	-	−0.075 (−0.006–0.003)	0.452	-	−0.064 (−0.005–0.003)	0.543
Farm	0.036	-		0.500	-		0.670	-		0.002	-		0.002	-		0.001	-	
Market	0.020			0.413			0.215			0.000			0.000			0.000		
Pesticide use	-	−0.039 (−0.039–0.029)	0.777	-	0.521 (−0.085–1.080)	0.093	-	−0.096 (−0.946–0.754)	0.824	-	0.029 (−0.004–0.005)	0.833	-	−0.054 (−0.007–0.005)	0.005	-	−0.038 (−0.006–0.004)	0.792
Yes	0.040	-		0.456	-		0.692	-		0.000	-		0.001	-		0.001	-	
No	0.013			0.840			0.118			0.001			0.000			0.000		
Use of PPE	-	0.204 (0.008–0.086)	0.019	-	0.000 (−0.664–0.662)	0.998	-	0.748 (−0.220–1.715)	0.129	-	−0.044 (−0.007–0.004)	0.612	-	−0.231 (−0.016–0.002)	0.008	-	0.074 (−0.003–0.008)	0.415
Yes	0.007	-		0.392	-		0.256	-		0.010	-		0.106	-		0.000	-	
No	0.002			0.457			0.000			0.000			0.000			0.000		
R2	0.127			-	0.089		0.079			0.119			0.122			0.034		

Var, variables; M, mean; Est coeff, estimation coefficient (95% CI).

## Data Availability

The raw data supporting the conclusions of this article will be made available by the authors on request.

## Supplementary Materials

The following supporting information can be downloaded at: https://www.mdpi.com/article/10.3390/jox15040120/s1, Supplementary File S1, list of internal standards; and jox-15-00120, Supplementary File S2, general screening method and quality control.

## Abbreviations

The following abbreviations are used in this manuscript:

LC-MS/MS	Liquid chromatography-tandem mass spectrometry
IS	Internal standard
MRM	Multiple reaction monitoring
LOD	Limit of detection
PLS-DA	Partial least squares-discriminant analysis
PPE	Personal protective equipment
WHO	World Health Organization
EDCs	Endocrine disruptors
