# Supplementary material for: From Farmworkers to Urban Residents: Mapping Multi-Class Pesticide Exposure Gradients in Morocco via Urinary Biomonitoring
Source: J Xenobiot. 2025 Jul 23;15(4):120. doi: 10.3390/jox15040120 (PMC12387287; doi:10.3390/jox15040120)
Supplement: Supplementary file 1 [file jox-15-00120-s001.zip › jox-3727112 Supplementary File S2.pdf]

General screening method and quality control

The critical aspects of quality assurance and quality control (QA/QC) in our analytical methodology. We have now included a detailed description of the steps undertaken to ensure accuracy, reproducibility, and recovery efficiency in both LC-MS/MS and GC-MS/MS analyses.

1. Quality Assurance and Instrumental Calibration

Each analytical sequence included calibration curves prepared in matrix-matched conditions (urine or serum) spanning the range of 0.01–10 µg/L. The method employed both quantifier and qualifier ion transitions in MRM mode, and a compound was considered positively identified only when the qualifier/quantifier ion ratio matched the standard within ±20% tolerance, in accordance with EU SANTE/11312/2021 guidelines. The instruments were tuned daily, and retention times were monitored to stay within ±0.1 min of standard references.

2. Quality Control and Replicates

All analyses were conducted in analytical replicates, and the method included low (LDQ), medium, and high concentration quality controls, each analyzed in 6 replicates (repeta) and on 6 different days (repro) to assess repeatability and intermediate precision.

These values are within acceptable limits for environmental biomonitoring assays, and all %RSDs have been provided in Supplementary Table S1.

3. Matrix Effects and Sensitivity

Matrix-matched calibration curves were used to correct for ion suppression/enhancement effects. Method detection limits (MDLs) and quantification limits (LOQs) were determined according to ICH Q2(R1), and ranged from 0.01 µg/L (LOQ) with signal-to-noise ratio ≥10.

Calibration curves were matrix-match built with concentrations ranging from 0,01 µg/L to 10 µg/L upon the sensitivity of the molecule.

Table S1. Repeatability and reproducibility (CV%) for all compounds across concentration levels.

	LDQ	LDQ	Med	Med	High	High
Substances	RPT CV %	RPO CV%	RPT CV %	RPO CV%	RPT CV %	RPO CV%
1-ANA	4,4	10,3	3,3	6,0	3,7	3,9
1-NAD	3,5	9,4	1,0	2,7	2,8	2,0

	LDQ	LDQ	Med	Med	High	High
Substances	RPT CV %	RPO CV%	RPT CV %	RPO CV%	RPT CV %	RPO CV%
2,4 D	9,8	24,5	10,2	10,2	3,4	5,3
2,4,6-TCP	10,7	11,3	4,7	12,5	7,6	9,9
2.4 DMA	5,9	5,1	5,4	5,1	4,4	6,6
Abamectin	7,6	3,1	5,2	10,4	4,2	14,5
Acephate	3,2	10,6	3,3	3,2	4,2	4,6
Acetamiprid	2,6	6,0	2,1	2,7	4,4	4,8
Acetochlor	1,9	8,0	2,3	3,3	2,1	3,7
Acibenzolar acid	16,2	15,1	10,5	9,8	5,9	11,3
Acibenzolar-S-methyl	7,3	23,9	3,5	5,5	5,4	4,5
Aclonifen	4,6	20,1	4,7	4,0	2,3	4,0
Acrinathrine	2,8	8,8	4,9	8,6	2,0	7,2
Aldicarb	3,2	8,1	4,0	2,6	4,9	6,1
Aldicarb sulfone	2,5	13,0	4,9	5,6	4,4	8,2
Aldicarb sulfoxide	3,3	8,4	6,6	5,2	5,7	5,0
Aldrin	6,6	9,3	6,8	7,7	0,5	1,9
Amectotradin	1,9	10,6	4,5	6,7	3,8	4,5
Ametryn	3,8	7,9	4,0	3,9	4,0	6,0
Amidosulfuron	3,1	15,5	3,4	6,1	4,3	3,4
Amisulbrom	16,9	8,8	7,6	8,2	3,8	7,1
Atrazine	0,0	8,5	1,4	4,5	2,9	4,0
Atrazine-2-hydroxy	3,2	14,7	6,4	3,6	3,0	3,6
Atrazine-desethyl	3,2	12,3	2,6	5,0	4,3	4,2
Atrazine-desisopropyl	3,2	6,5	2,6	3,8	5,5	6,3
Azadirachtin	5,3	21,2	5,0	3,9	5,2	9,1
Azimsulfuron	8,5	22,0	2,7	6,2	5,5	4,9
Azinphos-ethyl	7,0	6,9	5,0	6,4	0,9	0,8
Azinphos-methyl	3,4	14,3	6,3	3,7	6,6	9,2
Azoxystrobin	4,7	10,8	4,5	5,1	4,8	3,9
Bendiocarb	8,0	10,8	4,2	7,7	6,3	10,6
Benfluralin	4,7	6,8	2,7	3,5	2,4	8,3
Bentazone	15,6	12,5	12,4	9,3	7,5	17,5
Bentazone-8-OH	19,1	8,5	11,1	7,9	5,4	10,8
Benthiavalicarb-isopropyl	2,9	9,9	5,1	6,4	4,1	4,6
Benzyladenine	11,5	6,5	12,2	7,8	5,4	12,9
Bifenazate	4,5	20,2	5,7	7,5	4,3	7,2
Bifenox	7,4	16,0	8,2	11,6	4,7	4,5
Bifenthrine	3,2	13,7	5,4	5,3	2,7	8,2
Bitertanol	6,9	21,2	3,6	13,8	8,0	14,2
Bixafen	5,0	15,2	6,9	6,9	3,7	4,2
Boscalid	2,1	16,0	4,7	7,1	5,4	5,7
Brodifacoum	7,6	3,1	5,2	10,4	4,2	14,5
Bromacil	4,6	10,3	2,1	3,3	4,0	3,5

	LDQ	LDQ	Med	Med	High	High
**Substances**	**RPT CV %**	**RPO CV%**	**RPT CV %**	**RPO CV%**	**RPT CV %**	**RPO CV%**
Bromadiolone	2,7	10,1	5,9	7,0	13,0	9,0
Bromophos	3,3	9,3	6,5	7,1	10,7	11,5
Bromopropylate	3,7	14,1	5,8	5,5	3,1	8,3
Bupirimate	2,8	11,2	3,5	4,2	5,3	4,2
Buprofezin	2,2	6,3	4,7	4,8	9,5	9,7
Cadusafos	1,1	4,2	1,5	2,1	0,8	3,4
Carbaryl	3,3	10,6	3,9	5,1	4,7	3,4
Carbendazim	1,4	3,9	1,9	3,2	2,0	2,6
Carbetamide	4,9	11,7	3,0	2,3	4,1	3,2
Carbofuran	2,7	8,6	4,3	4,3	12,1	12,0
Carbofuran-3-hydroxy	3,9	22,9	2,2	3,7	3,5	4,0
Carboxin	5,9	30,1	10,5	11,0	9,2	15,9
Chlorantraniliprole	5,7	24,7	3,0	8,2	4,4	4,3
Chlordane-cis	13,0	14,2	7,9	6,5	3,9	2,2
Chlordane-trans	7,9	6,5	10,8	8,0	1,4	1,3
Chlordecone	11,1	9,0	6,8	5,4	1,4	2,4
Chlorfenapyr	7,3	20,7	4,9	4,1	3,1	6,8
Chlorfluazuron	8,0	19,3	6,8	8,9	5,4	7,2
Chlorobenzilate	2,9	4,5	2,2	5,8	1,0	2,1
Chlorophacinone	3,0	15,6	4,6	3,9	2,8	6,8
Chlorothalonil	9,9	5,7	0,8	5,0	0,8	4,0
Chlorpropham	1,7	7,6	1,3	3,2	1,8	3,3
Chlorpyrifos-methyl	1,9	3,8	1,6	2,8	1,4	3,4
Chlorpyriphos ethyl	2,4	3,3	0,6	2,1	1,1	1,9
Clodinafop	7,4	19,8	4,2	8,4	3,4	6,0
Clofentezine	2,8	8,0	5,5	5,1	4,8	4,3
Clomazone	0,7	7,5	1,5	2,1	1,6	2,2
Clothianidin	2,0	1,8	4,3	2,3	2,9	3,8
Coumachlor	3,0	10,2	1,9	4,8	3,6	4,4
Coumaphos	1,9	10,1	0,8	2,0	1,2	2,1
Coumatetralyl	10,2	32,2	9,8	15,4	0,5	3,4
Crimidine	3,7	7,6	3,3	3,2	1,8	3,4
Cyantraniliprole	9,4	12,8	1,7	6,8	2,6	5,3
Cyazofamid	3,9	12,7	6,3	8,2	4,6	4,9
Cycloxydim	14,9	24,5	8,0	6,7	4,8	10,2
Cyflufenamid	3,6	12,6	5,7	3,3	3,2	4,0
Cyflumetofen	2,6	11,7	6,6	8,1	2,7	6,0
Cyfluthrine	3,1	10,7	5,5	7,8	2,3	7,3
Cymoxanil	3,3	24,2	5,5	5,4	6,8	6,5
Cypermethrine	6,8	10,9	5,4	8,9	2,7	7,9
Cyproconazole	4,2	10,8	3,9	5,5	5,0	4,4
Cyprodinil	5,9	7,1	2,8	4,2	4,4	3,5

	LDQ	LDQ	Med	Med	High	High
**Substances**	**RPT CV %**	**RPO CV%**	**RPT CV %**	**RPO CV%**	**RPT CV %**	**RPO CV%**
Cyromazine	2,5	4,4	1,3	1,5	0,6	3,1
DDE-o,p'	2,4	6,2	2,5	3,4	1,4	1,7
DDE-p,p'	4,6	12,6	7,9	5,2	1,2	5,6
DDT-o,p'	10,6	14,0	5,7	5,9	1,6	2,6
DDT-p,p'	7,9	10,0	5,6	4,8	0,5	1,4
DEET	3,9	9,2	3,3	2,9	10,6	7,8
Deltamethrine	4,9	13,2	5,1	8,5	3,0	5,4
Desmedipham	2,7	10,1	5,9	7,0	13,0	9,0
Diazinon	3,3	9,3	6,5	7,1	10,7	11,5
Dichlorprop	4,9	7,4	7,8	3,0	6,2	7,3
Dichlorvos	8,1	8,2	3,1	6,1	3,1	3,5
Dicloran	2,6	3,2	2,7	4,1	1,5	3,1
Dicofol	4,4	15,9	6,6	7,9	4,0	14,0
Dieldrin	2,8	4,5	13,7	11,5	0,7	1,3
Difenacoum	3,3	9,8	2,5	4,5	4,9	3,5
Difenoconazole	1,1	8,7	5,0	3,8	4,1	2,6
Diflubenzuron	2,8	16,2	6,0	6,0	3,3	5,8
Diflufenican	2,6	16,3	5,5	4,4	2,8	8,0
Dimethachlor	5,9	6,4	4,9	8,3	2,9	8,9
Dimethoate	3,8	8,3	2,6	4,0	4,5	5,4
Dimethomorph	3,3	11,6	3,9	4,6	3,8	4,2
Dimoxystrobin	20,9	24,7	7,5	3,7	5,0	11,0
Dinocap	10,9	12,7	5,0	7,4	3,6	9,8
Dinotefuran	4,4	17,4	2,2	3,4	3,9	3,4
Diuron	2,6	10,2	2,6	2,7	4,9	4,1
DMF	5,1	7,0	1,6	4,0	3,7	4,2
DMPF	5,3	9,1	2,2	2,9	3,8	4,5
DMST	5,5	10,2	3,8	6,2	4,5	4,7
Dodine	6,1	16,2	5,7	7,0	5,7	5,0
Emamectin B1a	3,9	12,6	4,2	4,1	3,4	2,8
Emamectin B1b	13,1	23,9	3,7	8,0	13,2	17,9
Endosulfan alpha	4,3	9,0	1,3	2,7	1,9	3,8
Endosulfan beta	4,7	11,2	4,7	4,1	2,8	7,7
Endosulfan ether	3,9	7,5	4,9	6,6	12,3	3,6
Endosulfan lactone	11,3	15,0	3,1	7,5	1,0	2,3
Endosulfan sulfate	7,9	7,7	11,7	8,4	1,1	2,0
Epoxiconazole	2,7	8,7	4,0	4,8	4,4	3,3
Ethiofencarb	3,4	12,1	5,6	7,7	3,0	7,3
Ethion	3,5	13,2	3,7	2,7	2,3	6,2
Ethofenprox	3,9	13,2	5,6	7,9	3,0	7,4
Ethofumesate	3,2	11,1	3,4	2,4	2,2	4,6
Ethofumesate, 2-keto-	6,1	20,7	5,2	4,2	3,8	4,9

	LDQ	LDQ	Med	Med	High	High
**Substances**	**RPT CV %**	**RPO CV%**	**RPT CV %**	**RPO CV%**	**RPT CV %**	**RPO CV%**
Ethoprophos	2,3	10,2	1,9	2,7	0,8	4,3
Etoxazole	9,3	28,0	12,8	14,9	13,6	18,9
Famoxadone	4,4	14,1	6,4	8,2	4,4	6,6
Fenamiphos	7,7	11,5	5,4	8,6	5,6	5,3
Fenamiphos sulfone	5,0	6,7	3,4	3,9	2,7	4,7
Fenamiphos sulfoxide	3,3	9,0	2,5	4,5	4,9	3,5
Fenarimol	4,7	15,8	3,3	6,5	4,0	3,8
Fenazaquin	2,1	12,9	6,1	5,7	5,0	5,2
Fenbuconazole	2,3	9,7	4,2	3,7	3,4	2,7
Fenbutatin oxide	7,4	12,9	8,3	6,4	2,9	4,6
Fenhexamid	5,8	9,5	3,3	3,7	4,6	4,8
Fenitrothion	1,9	10,1	0,8	2,0	1,2	2,1
Fenobucarb	9,2	32,2	9,8	20,2	0,5	3,4
Fenoxaprop-P	22,2	15,0	6,9	5,0	7,1	5,8
Fenoxycarb	2,8	12,3	4,9	6,2	4,2	4,7
Fenpropathrine	3,7	13,0	5,6	6,1	2,6	7,8
Fenpropidin	3,2	12,2	4,0	3,0	4,1	5,0
Fenpropimorph	3,7	12,4	4,3	4,8	4,5	4,3
Fenpyrazamine	4,3	10,5	4,9	4,6	3,9	3,7
Fenpyroximate	2,4	9,6	7,0	6,6	5,2	6,9
Fenthion	4,1	7,9	7,3	6,3	6,2	6,0
Fenthion sulfone	13,5	10,7	4,7	4,6	5,2	5,7
Fenthion sulfoxide	4,3	11,6	2,2	6,2	3,5	6,0
Fenvalerate	7,8	17,5	5,5	5,2	3,3	8,4
Fipronil	13,3	10,6	7,4	6,0	6,1	11,2
Fipronil sulfone	17,2	19,1	6,8	6,9	4,1	10,3
Flazasulfuron	4,0	12,4	5,2	3,6	4,1	4,2
Flocoumafen	13,9	13,2	6,0	7,8	0,8	2,5
Flonicamid	19,0	9,5	12,9	9,8	5,4	11,4
Florasulam	3,4	13,7	4,0	5,6	5,2	5,2
Fluazinam	12,9	14,4	8,1	7,5	5,6	12,0
Flubendiamide	24,7	41,2	10,2	17,5	16,2	24,1
Fludioxonil	15,4	16,1	9,5	6,6	4,8	10,2
Flufenacet	3,9	14,1	4,5	4,1	2,7	6,1
Flufenoxuron	2,5	16,3	9,2	6,4	5,3	5,3
Flumioxazin	6,0	13,7	5,8	8,0	4,3	5,3
Fluopicolid	9,3	4,4	7,2	7,3	2,1	2,4
Fluopyram	3,2	14,4	5,4	4,2	4,1	5,3
Fluoxastrobin	3,3	11,2	6,1	5,5	3,9	4,0
Flupyrsulfuron-methyl	3,8	12,7	5,4	6,9	4,1	4,7
Fluquinconazole	5,3	13,1	4,7	4,8	4,6	4,5
Fluroxypyr-1-methylheptyl	5,2	17,7	5,5	4,9	2,6	7,7

Substances	LDQ RPT CV %	LDQ RPO CV%	Med RPT CV %	Med RPO CV%	High RPT CV %	High RPO CV%
Flurtamone	2,6	11,7	5,7	4,8	3,1	4,6
Flusilazole	1,9	11,0	4,9	4,5	5,2	3,0
Flutriafol	3,0	15,1	5,3	6,2	4,3	4,6
Fluvalinate	4,6	13,5	5,0	7,5	2,6	6,5
Fluxapyroxad	0,7	6,9	1,0	2,1	1,4	2,2
Fonophos	2,5	4,5	1,3	0,7	0,9	2,9
Foramsulfuron	7,9	16,2	2,4	5,9	1,7	5,6
Forchlorfenuron	4,9	12,1	4,2	5,6	4,3	4,3
Formetanate HCl	2,9	7,9	4,0	3,4	6,4	7,1
HCH alpha	4,6	7,4	2,9	9,6	0,7	2,0
HCH beta	9,7	6,6	4,6	4,6	0,8	1,4
HCH delta	7,7	6,2	1,6	4,1	0,7	1,8
HCH gamma	6,6	5,3	2,8	9,6	1,8	8,1
Heptachlor	2,6	8,1	2,9	7,5	2,3	3,7
Heptachlor exo-epoxide	5,7	5,2	5,8	5,7	0,5	1,8
Hexachlorobenzene	8,3	10,2	2,2	2,0	0,5	1,8
Hexaconazole	5,8	23,0	8,0	11,8	8,0	8,8
Hexythiazox	3,6	12,8	6,8	6,5	4,5	4,3
Hydramethylnon	3,5	9,1	7,8	6,0	4,6	5,1
Imazalil	3,8	9,6	1,4	0,8	2,4	3,5
Imazamox	5,1	9,9	3,5	4,4	4,0	2,7
Imidacloprid	4,1	10,4	2,6	4,1	4,0	4,5
Indaziflame	4,2	12,7	4,8	3,8	6,7	12,4
Indoxacarb	4,7	8,2	7,7	5,4	5,6	4,8
Iodosulfuron-methyl	6,2	24,4	8,0	4,5	6,3	5,9
Ipconazole	4,2	10,8	3,9	5,5	5,0	4,4
Iprodione	6,5	20,5	5,8	5,3	3,1	7,6
Iprovalicarb	5,9	7,1	2,8	4,2	4,4	3,5
Isobenzan	2,0	18,2	4,0	12,3	0,5	1,7
Isofenphos	2,9	8,1	2,1	3,0	2,2	4,7
Isoprocarb	2,5	4,4	1,3	1,5	0,6	3,1
Isoproturon	1,4	6,7	3,1	2,2	1,5	0,6
Isopyrazam	2,4	6,2	2,5	3,4	1,4	1,7
Isoxaben	2,9	13,0	7,1	4,5	6,8	6,5
Isoxadifen-ethyl	3,1	15,1	5,2	4,1	2,4	6,3
Kresoxim methyl	3,9	14,2	4,0	4,5	3,9	4,5
Lambda cyhalothrine	2,2	14,6	5,2	7,1	2,6	7,3
Lufenuron	19,6	46,4	7,5	19,2	11,3	10,6
Malaoxon	3,2	8,1	4,0	2,6	4,9	6,1
Malathion	2,5	13,0	4,9	5,6	4,4	8,2
Mandipropamid	3,3	8,4	6,6	5,2	5,7	5,0
MCPA	7,0	6,9	5,0	6,4	0,9	0,8

	LDQ	LDQ	Med	Med	High	High
**Substances**	**RPT CV %**	**RPO CV%**	**RPT CV %**	**RPO CV%**	**RPT CV %**	**RPO CV%**
Mecarbam	4,7	6,8	2,7	3,5	2,4	8,3
Mecoprop	20,5	10,5	10,5	6,7	4,1	10,8
Mefenoxam	3,6	3,4	0,9	4,2	2,2	6,4
Mepanipyrim	3,1	9,4	4,0	4,5	4,1	3,8
Mephosfolan	3,0	6,6	2,3	2,4	4,5	4,7
Mesosulfuron methyl	5,2	17,6	3,2	5,3	3,8	4,0
Mesotrione	7,9	12,9	3,2	8,0	3,8	4,7
Metalaxyl	2,2	8,1	3,7	4,2	6,6	6,1
Metaldehyde	14,7	36,9	4,7	11,3	3,7	3,6
Metamitron	3,9	12,5	1,6	3,6	3,2	4,2
Metazachlor	7,9	7,7	11,7	8,4	1,1	2,0
Metconazole	3,6	15,0	4,5	5,2	4,4	5,3
Methamidophos	3,4	8,8	3,0	2,7	4,4	4,7
Methidathion	3,9	7,2	5,8	6,9	5,3	6,7
Methiocarb	4,2	18,9	5,3	6,7	3,9	6,5
Methiocarb sulfone	4,2	7,9	3,3	5,5	4,0	4,6
Methiocarb sulfoxide	2,9	7,9	3,1	3,9	8,0	7,5
Metholachlor	3,4	12,1	5,6	7,7	3,0	7,3
Methomyl	2,5	5,2	2,7	3,7	1,5	6,0
Methoxyfenozide	6,9	9,2	4,9	6,9	4,8	5,3
Metobromuron	7,4	14,6	3,9	6,4	4,3	3,7
Metolcarb	6,4	4,7	4,1	7,3	1,7	3,1
Metrafenone	11,2	25,9	5,3	13,8	4,6	6,1
Metribuzin	6,8	22,5	11,2	23,0	9,4	11,9
Metsulfuron methyl	2,2	19,8	2,7	4,6	4,2	4,5
Mevinphos	9,3	28,0	12,8	14,9	13,6	18,9
Mirex	1,2	9,5	0,7	6,4	0,4	2,6
Myclobutanil	3,8	15,5	3,9	6,5	5,5	5,7
Napropamide	2,0	18,0	5,4	4,7	3,1	4,7
Nicosulfuron	3,1	13,4	3,0	5,8	4,6	5,2
Nonachlor, trans-	10,4	13,6	12,8	13,5	1,0	3,4
Norflurazon	4,7	10,9	2,0	3,1	0,7	2,5
Novaluron	3,8	15,9	5,2	6,3	5,4	6,2
Nuarimol	8,9	13,9	5,2	7,5	4,9	5,9
Omethoate	4,2	9,3	2,9	3,5	4,1	3,6
Oryzalin	22,9	23,2	8,5	7,5	6,9	14,6
Oxadiazon	3,4	12,9	5,1	4,1	3,1	6,8
Oxadixyl	14,4	9,5	2,4	6,6	3,1	5,9
Oxamyl	3,8	13,2	2,4	5,8	3,1	5,3
Oxydemeton methyl	3,1	9,3	3,3	3,8	6,0	6,5
Oxydemeton methyl sulfone	3,4	10,9	3,2	4,3	3,0	3,4
Oxyfluorfen	2,8	14,1	4,2	3,1	2,2	3,5

	LDQ	LDQ	Med	Med	High	High
**Substances**	**RPT CV %**	**RPO CV%**	**RPT CV %**	**RPO CV%**	**RPT CV %**	**RPO CV%**
Paclobutrazol	7,7	13,6	4,5	3,0	2,5	5,2
Parathion-methyl	2,2	5,5	1,3	3,7	0,9	2,6
Penconazole	5,1	5,3	4,1	4,3	3,5	4,9
Pencycuron	2,4	9,7	4,7	4,8	3,0	3,6
Pendimethaline	4,5	6,9	1,3	2,0	0,9	1,7
Penoxsulam	1,9	11,5	5,4	5,8	4,5	5,3
Penthiopyrad	2,4	11,8	5,1	5,8	2,6	7,5
Permethrin	10,4	3,3	7,0	6,0	1,2	1,7
Phenmedipham	3,4	10,8	5,1	5,9	2,7	5,4
Phenyl-2-phenol	24,7	6,7	4,1	8,2	4,0	5,1
Phorate	11,8	8,6	4,6	7,7	5,0	13,0
Phorate sulfone	2,7	4,2	1,8	2,9	2,4	5,5
Phorate sulfoxide	3,4	10,1	3,9	3,9	4,9	2,8
Phosalone	3,9	11,6	3,4	5,3	3,5	4,3
Phosmet	2,5	9,1	4,6	3,5	4,0	4,5
Phosmet oxon	3,9	14,2	4,0	4,5	3,9	4,5
Phoxim	3,5	11,7	5,6	3,9	4,0	4,1
Phthalimide	24,3	18,4	5,7	9,5	3,0	3,7
Picolinafen	3,5	13,2	3,7	2,7	2,3	6,2
Picoxystrobin	3,9	13,2	5,6	7,9	3,0	7,4
Pinoxaden	3,3	8,9	5,3	5,3	5,6	7,4
Piperonyl butoxide	2,8	7,5	5,5	4,4	4,8	10,9
Pirimicarb	3,6	10,1	2,9	3,5	4,4	5,0
Pirimicarb-desmethyl	3,2	11,1	3,4	2,4	2,2	4,6
Pirimiphos-ethyl	6,1	20,7	5,2	4,2	3,8	4,9
Pirimiphos-methyl	2,3	10,2	1,9	2,7	0,8	4,3
Prochloraz	1,7	8,1	3,7	4,4	4,1	3,5
Procymidone	4,3	17,9	4,1	4,1	2,7	4,8
Profenofos	3,1	7,4	6,0	3,6	3,5	2,4
Prohexadione Ca	4,0	13,3	5,1	1,5	2,7	6,2
Promecarb	4,4	14,1	6,4	8,2	4,4	6,6
Prometryn	7,7	11,5	5,4	8,6	5,6	5,3
Propamocarb	3,7	10,8	2,1	2,7	4,2	4,2
Propaquizafop	4,2	11,8	6,7	7,2	4,3	4,4
Propargite	3,5	10,4	7,4	6,6	4,6	4,7
Propiconazole	6,0	21,9	6,5	1,6	4,4	6,0
Propoxur	2,7	8,7	4,0	4,8	4,4	3,3
Propyzamide	2,9	11,3	4,5	8,0	2,2	3,2
Proquinazid	5,0	6,7	3,4	3,9	2,7	4,7
Prosulfocarb	2,5	2,4	1,0	2,7	1,3	2,3
Prosulfuron	3,7	16,2	5,7	13,8	7,1	11,3
Prothioconazole	17,2	24,5	10,3	5,5	8,3	19,7

Substances	LDQ<br>RPT CV %	LDQ<br>RPO CV%	Med<br>RPT CV %	Med<br>RPO CV%	High<br>RPT CV %	High<br>RPO CV%
Prothioconazole desthio	2,8	24,7	3,7	4,5	4,6	6,3
Pymetrozine	1,9	10,4	3,9	5,3	5,2	5,5
Pyraclostrobin	3,1	8,0	5,5	4,9	4,0	3,2
Pyrazophos	2,5	7,6	7,1	3,2	8,1	8,1
Pyridaben	3,4	11,4	7,4	8,3	5,9	5,9
Pyridafol	2,8	15,3	1,7	4,0	3,6	4,4
Pyridalyl	3,4	15,7	6,8	9,9	2,8	6,9
Pyridate	3,0	14,5	8,5	4,6	3,8	4,6
Pyrifenox	3,1	9,2	2,5	3,4	4,3	2,3
Pyrimethanil	3,7	5,4	2,0	3,9	4,5	3,6
Pyriproxyfen	3,4	10,9	7,1	5,7	11,1	10,0
Quinalphos	2,6	11,8	4,8	5,2	4,8	4,1
Quinmerac	3,4	12,2	2,2	6,2	3,1	4,8
Quinoxyfen	2,1	14,4	3,3	5,9	4,2	4,4
Quizalofop-ethyl	5,9	11,9	5,3	9,8	3,3	6,6
Rotenone	4,2	14,6	5,2	5,5	4,5	3,4
Silthiofam	3,8	18,4	5,8	13,3	2,6	5,1
Spinetoram J	11,7	11,6	7,1	7,7	7,6	9,2
Spinetoram L	3,8	10,3	5,9	4,1	2,6	5,3
Spinosad A	2,4	9,7	4,7	4,8	3,0	3,6
Spinosad D	8,6	12,0	5,8	5,2	5,8	5,3
Spirodiclofen	4,1	13,7	5,2	8,1	3,2	8,2
Spiromesifen	3,5	24,6	6,5	7,9	1,5	8,1
Spirotetramat	6,7	9,3	5,6	5,4	4,4	4,4
Spirotetramat<br>cis keto hydroxy	8,5	22,0	2,7	6,2	5,5	4,9
Spirotetramat enol	2,6	12,2	3,3	6,1	3,5	4,6
Spirotetramat enol glucoside	7,6	16,5	3,4	4,6	3,8	3,7
Spirotetramat mono hydroxy	3,8	19,4	1,4	3,0	3,5	4,3
Spiroxamine	3,5	7,6	4,2	1,6	9,5	9,9
Sulcotrione	3,1	8,7	2,4	4,6	3,3	3,6
Sulfosulfuron	3,0	11,0	3,1	6,4	2,7	3,3
Tebuconazole	2,5	10,4	4,8	6,8	5,1	6,3
Tebufenozide	3,8	15,1	6,4	8,4	5,5	3,7
Tebufenpyrad	1,9	15,9	4,3	4,8	4,4	5,8
Teflubenzuron	8,2	15,5	4,1	16,3	7,1	12,6
Tembotrione	7,4	16,1	5,4	7,5	5,7	4,0
Terbufos	6,0	6,3	3,8	5,1	3,7	9,3
Terbuthylazine	12,2	8,5	10,8	1,9	5,5	3,1
Terbutryn	4,4	10,3	3,3	6,0	3,7	3,9
Tetraconazole	1,0	9,2	5,1	6,6	4,9	5,2
Tetradifon	4,5	15,1	6,8	7,0	3,8	7,1

	LDQ	LDQ	Med	Med	High	High
**Substances**	**RPT CV %**	**RPO CV%**	**RPT CV %**	**RPO CV%**	**RPT CV %**	**RPO CV%**
TFNA	24,0	14,3	14,1	6,9	6,1	10,5
TFNG	3,2	14,7	6,4	3,6	3,0	3,6
Thiabendazole	3,2	12,3	2,6	5,0	4,3	4,2
Thiacloprid	3,2	6,5	2,6	3,8	5,5	6,3
Thiamethoxam	4,4	11,4	2,4	2,6	2,6	2,6
Thiencarbazone-methyl	5,2	14,6	4,3	2,3	5,8	4,4
Thifensulfuron-methyl	3,3	11,8	2,4	4,1	4,0	4,3
Thiodicarb	2,2	12,2	3,7	4,0	4,6	3,8
Thiophanate methyl	0,9	13,1	4,8	7,8	5,4	8,9
THPI	4,3	11,1	4,1	4,3	3,4	3,5
Triadimefon	3,1	11,9	4,1	4,6	4,7	4,0
Triadimenol	3,6	6,4	3,5	4,5	4,5	5,7
Triallate	33,3	35,8	6,3	14,9	6,1	4,2
Tribenuron-methyl	16,9	8,8	7,6	8,2	3,8	7,1
Trichlorfon	0,0	8,5	1,4	4,5	2,9	4,0
Triclopyr	2,3	13,0	2,1	1,9	2,1	3,9
Tridemorph	5,7	11,7	4,7	2,8	4,7	5,3
Trifloxystrobin	2,5	7,6	7,1	3,2	8,1	8,1
Triflumizole	2,3	6,8	4,7	4,0	4,8	3,9
Triflumuron	3,3	9,3	5,8	4,1	6,1	7,5
Triforine	10,7	20,8	7,4	7,4	4,2	5,6
Trinexapac	9,2	14,6	3,5	5,8	4,0	2,9
Triticonazole	12,8	8,9	4,6	5,0	4,6	3,9
Tritosulfuron	9,5	12,3	6,3	4,0	4,3	3,1
Valifenalate	4,1	9,6	5,3	5,3	4,4	3,2
Vamidothion	2,5	6,2	2,5	3,0	7,0	7,9
Vinclozolin	3,1	6,6	1,1	1,6	1,3	2,3
Warfarin	5,8	6,2	3,5	9,4	1,6	3,1
Zoxamide	10,0	15,6	10,2	11,2	4,4	5,9

Med, medium; RPT, repetability; RPO, reproducibility; CV, coefficient of variation.
